# Does Inflammation Mediate the Association Between Sleep Duration and Incident Dementia Among Older Adults?

**DOI:** 10.1093/geroni/igaa057.391

**Published:** 2020-12-16

**Authors:** Chenkai Wu, Weihao Xu

**Affiliations:** 1 Duke Kunshan University, Kunshan, Jiangsu, China; 2 Chinese PLA General Hospital, Beijing, China

## Abstract

Sleep duration is a risk factor for multiple health outcomes. Growing attention has been directed to the association between sleep duration and dementia; however, results were inconsistent and the mechanisms remained largely unknown. We hypothesized that elevated levels of inflammation markers— C reactive protein (CRP), interleukin-6 (IL-6), and tumor necrosis factor-α (TNF-α)—would mediate the association between sleep duration and dementia among older adults. Data were from the Health, Aging, and Body Composition Study; 3,010 participants free of dementia at baseline were included. Sleep duration was classified into: short (<6 hours), normal (6-8 hours), and long (>8 hours). Incident dementia was defined as (i) use of prescribed dementia medications, (ii) adjudicated dementia diagnosis, or (iii) a race-stratified cognitive decline >1.5 SDs from the baseline mean. We used Cox models to examine the associations among sleep duration, inflammation, and dementia. The average age was 73.6 years (SD=2.9); 49% were male and 41% were black. During 10 years of follow-up, 515 participants (17.1%) developed dementia. Long sleep duration was associated with higher hazard of dementia than normal sleep duration (HR=1.50, 95%CI=1.02-2.21). This association was attenuated by approximately 10% when CRP or IL-6 was added in the model. When all three inflammation markers were included in the model, the hazard ratio of long sleep duration was reduced by nearly 30% and no longer significant (HR=1.36, 95%CI=0.89-2.08). Long sleep duration was associated with high risk of incident dementia among older adults and the association was partly explained by elevated levels of inflammation markers.

